# Quinones and Aromatic Chemical Compounds in Particulate Matter Induce Mitochondrial Dysfunction: Implications for Ultrafine Particle Toxicity

**DOI:** 10.1289/ehp.7167

**Published:** 2004-07-07

**Authors:** Tian Xia, Paavo Korge, James N. Weiss, Ning Li, M. Indira Venkatesen, Constantinos Sioutas, Andre Nel

**Affiliations:** ^1^Division of Clinical Immunology and Allergy, Department of Medicine; ^2^The Southern California Particle Center and Supersite; ^3^Department of Physiology and Division of Cardiology, Department of Medicine, and; ^4^Institute of Geophysics and Planetary Physics, University of California, Los Angeles, California, USA; ^5^Department of Civil and Environmental Engineering, University of Southern California, Los Angeles, California, USA

**Keywords:** apoptosis, DEPs, diesel exhaust particles, PAHs, permeability transition pore, polycyclic aromatic hydrocarbons, quinones, ultrafine particles

## Abstract

Particulate pollutants cause adverse health effects through the generation of oxidative stress. A key question is whether these effects are mediated by the particles or their chemical compounds. In this article we show that aliphatic, aromatic, and polar organic compounds, fractionated from diesel exhaust particles (DEPs), exert differential toxic effects in RAW 264.7 cells. Cellular analyses showed that the quinone-enriched polar fraction was more potent than the polycyclic aromatic hydrocarbon (PAH)–enriched aromatic fraction in O_2_^•−^ generation, decrease of membrane potential (ΔΨm), loss of mitochondrial membrane mass, and induction of apoptosis. A major effect of the polar fraction was to promote cyclosporin A (CsA)–sensitive permeability transition pore (PTP) opening in isolated liver mitochondria. This opening effect is dependent on a direct effect on the PTP at low doses as well as on an effect on ΔΨm at high doses in calcium (Ca^2+^)-loaded mitochondria. The direct PTP effect was mimicked by redox-cycling DEP quinones. Although the aliphatic fraction failed to perturb mitochondrial function, the aromatic fraction increased the Ca^2+^ retention capacity at low doses and induced mitochondrial swelling and a decrease in ΔΨm at high doses. This swelling effect was mostly CsA insensitive and could be reproduced by a mixture of PAHs present in DEPs. These chemical effects on isolated mitochondria could be reproduced by intact DEPs as well as ambient ultrafine particles (UFPs). In contrast, commercial polystyrene nanoparticles failed to exert mitochondrial effects. These results suggest that DEP and UFP effects on the PTP and ΔΨm are mediated by adsorbed chemicals rather than the particles themselves.

There is increasing evidence that particulate pollutants induce inflammatory responses in the cardiorespiratory system (Nel et al. 1998; Nightingale et al. 2000; Saldiva et al. 2002). These proinflammatory effects have been linked to the ability of particulate matter (PM), such as diesel exhaust particles (DEPs), to generate reactive oxygen species (ROSs) and oxidative stress in macrophages, bronchial epithelial cells, and lung microsomes (Gurgueira et al. 2002; Hiura et al. 1999; Kumagai et al. 1997; Nel et al. 2001). The pro-oxidative effects of the intact particles can be mimicked by organic chemical components extracted from these particles (Hiura et al. 1999; Kumagai et al. 1997; Li et al. 2002). The PM-induced oxidative stress response is a hierarchical event, which is characterized by the induction of antioxidant and cytoprotective responses at lower tiers of oxidative stress and by pro-inflammatory and cytotoxic responses at higher levels of oxidative stress (Li et al. 2002; Xiao et al. 2003).

Mitochondrial damage is a key event in PM-induced cytotoxicity (Hiura et al. 1999, 2000). The initial response to PM is a decrease in mitochondrial membrane potential (ΔΨm) and increased O_2_^•−^ production, followed by cytochrome *c* release and inner mitochondrial membrane damage (Hiura et al. 1999, 2000; Upadhyay et al. 2003). It is also of interest that the smallest and potentially most toxic ambient particles, ultrafine particles (UFPs), lodge inside damaged mitochondria (Li et al. 2003). UFPs have a physical diameter < 0.1 μm, which allows them to penetrate deep into the lung as well as into systemic circulation (Nemmar et al. 2002). Although it is still a matter of debate whether UFPs target the mitochondrion directly or enter the organelle secondary to oxidative damage (Li et al. 2003), PM-induced mitochondrial perturbation has important biologic effects, which include the initiation of apoptosis and decreased ATP production (Hiura et al. 2000). Although the particles themselves may play a role in mitochondrial damage, it has been demonstrated that the organic chemicals adsorbed on the particle surface mimic the effects of the intact particles (Hiura et al. 1999). These effects can also be reproduced by functionalized aromatic and polar chemical groups fractionated from DEPs by silica gel chromatography (Alsberg et al. 1985; Li et al. 2000). These compounds are toxicologically relevant because the aromatic fraction is enriched in polycyclic aromatic hydrocarbons (PAHs), whereas the polar fraction contains several oxy-PAH compounds, including quinones (Alsberg et al. 1985; Li et al. 2000). Quinones are able to redox cycle and to produce ROSs, whereas PAHs can be converted to quinones by cytochrome P450, epoxide hydrolase, and dihydrodiol dehydrogenase (Penning et al. 1999).

A key mitochondrial target for oxidizing chemicals is the permeability transition pore (PTP) (Jajte 1997; Susin et al. 1998; Zoratti and Szabo 1995). This calcium (Ca^2+^)-, voltage-, and pH-sensitive pore is permeant to molecules of < 1.5 kDa and opens in the mitochondrial inner membrane when matrix Ca^2+^ levels are increased, especially when accompanied by oxidative stress (Bernardi 1999; Kushnareva and Sokolove 2000; Zoratti and Szabo 1995). PTP opening causes massive *in vitro* mitochondrial swelling, outer membrane rupture, and release of proapoptotic factors such as cytochrome *c* (Susin et al. 1998). In addition, mitochondria become depolarized, causing inhibition of oxidative phosphorylation and stimulation of ATP hydrolysis. PTP opening is inhibited by cyclosporin A (CsA), which inhibits the peptidyl-prolyl *cis-trans* isomerase activity of cyclophilin D (Bernardi 1999). This has led to the proposal that PTP transition is mediated by a Ca^2+^-triggered conformational change of inner membrane proteins (Woodfield et al. 1998). However, although this model may explain the action of some PTP modulators, PTP open–close transitions are also regulated by physiologic factors, drugs, and chemicals (Jajte 1997; Kushnareva and Sokolove 2000). Walter et al. (2000) characterized endogenous ubiquinones that stimulate or inhibit pore function by means of a putative quinone binding site in the PTP.

The goal of our study was to clarify how redox-cycling DEP chemicals affect mitochondrial function, as well as to compare ambient UFPs with commercial nanoparticle effects on mitochondria. Aromatic, polar, and aliphatic chemical fractions, prepared by silica gel chromatography, were used to study CsA-sensitive mitochondrial swelling (PTP opening), ΔΨm, Ca^2+^ loading capacity, and mitochondrial respiration. We also compared isolated mitochondrial responses with perturbation of mitochondrial function in intact RAW 264.7 cells. Our data show that mitochondrial perturbation and induction of apoptosis by polar DEP chemicals involve CsA-sensitive PTP opening that can be mimicked by representative redox-cycling quinones present in DEPs. In contrast, the aromatic chemical fraction induced mostly CsA-insensitive mitochondrial swelling, which can be mimicked by a mixture of PAHs. Ambient UFPs induced a combination of aromatic and polar effects, whereas polystyrene nanoparticles were inactive.

## Materials and Methods

### Reagents.

Tetramethylrhodamine methyl ester (TMRM), propidium iodide (PI), sucrose, HEPES buffer salts, EGTA, ascorbic acid, succinate, malate, glutamate, carbonyl cyanide *m*-chlorophenylhydrazone (CCCP), alamethacin (Ala), and tetraphenylphosphonium chloride were from Sigma (St. Louis, MO). The annexin V–fluorescein isothio-cyanate (FITC) kit was obtained from Trevigen (Gaithersburg, MD). 3,3′-Dihexyl-oxabarbocyanine iodide (DiOC_6_), 10 *N*-nonylacridine orange (NAO), Calcium Green-5N, and hydroethidine (HE) were obtained from Molecular Probes (Eugene, OR). The PAH working standard (no. 8310) was purchased from Cerilliant Corporation (Round Rock, TX). All organic solvents used were of Fisher optima grade (Fisher Scientific, Hampton, NH), and the solid chemicals were of analytical reagent grade.

### Preparation of crude DEP extracts.

DEPs were obtained from M. Sagai (National Institute of Environment Studies, Tsukuba, Ibaraki, Japan). These particles were collected from a 4JB1-type light-duty, 2.74-L, four-cylinder Isuzu diesel engine (Isuzu Automobile Co., Tokyo, Japan) under a load of 6 kilogram meter onto a cyclone impactor (Kumagai et al. 1997). The particles were scraped from the glass fiber filters and stored as a powder under nitrogen gas. The particles consist of aggregates in which individual particles are < 1 μm in diameter. The chemical composition of these particles, including PAH and quinone analysis, has been previously described (Li et al. 2000). DEP methanol extracts were prepared by suspending 100 mg particles in 25 mL methanol, followed by sonication and centrifuging the suspension at 2,000 rpm for 10 min at 4°C (Hiura et al. 1999). The supernatant was transferred to a preweighed polypropylene tube and dried under nitrogen gas. The tube was reweighed to determine the methanol-extractable phase. The dried extract was dissolved in DMSO, and aliquots stored at −80°C in the dark.

### DEP fractionation by silica gel chromatography.

DEPs (1.2 g) were sonicated in 200 mL methylene chloride, and the extract was filtered with a 0.45-μm nylon filter in a Millipore filtration system (Li et al. 2000). The methylene chloride extract was concentrated by rotoevaporation, and asphaltenes (insoluble, aromatic chemicals with nitrogen, oxygen, and sulfur heteroatoms) were precipitated by adding 25 mL hexane and shaking. The contents were left overnight in the freezer and then centrifuged, and the supernatant was collected. The precipitate was washed twice with hexane, and the washings were combined with the first hexane extract, concentrated, and dried over anhydrous sodium sulfate. The extract thus prepared was subjected to gravity-fed silica gel column chromatography. Three columns (1.5 × 50 cm) were packed with 26 g activated silica gel between 1 cm anhydrous sodium sulfate and conditioned with hexane. The extract was split into three equal aliquots and applied to each column. Aliphatic, aromatic, and polar fractions were successively eluted at 1.5 mL/min with 70 mL hexane, 150 mL hexane:methylene chloride (3:2, vol/vol), and 90 mL methylene chloride:methanol (1:1, vol/vol), respectively. The elution of the aromatic fraction was monitored by ultraviolet light at 365 nm. The respective eluates were combined and concentrated by rotoevaporation and made up to 1 mL in a 4-mL graduated vial, the aliphatic fraction in hexane and the others in methylene chloride. The vials were tightly sealed with a silicone-lined cap and stored at −80°C until use. The weight of the fractions was determined in a microbalance after evaporating off the hexane or methylene chloride from a known sample volume. Alkanes in the aliphatic fraction were characterized by gas chromatography (Varian 3400 with an SPI injector; Varian Inc., Palo Alto, CA) equipped with a flame ionization detector and a DB-5 column (30 m, 0.25 mm inner diameter, 0.25 μm film). The fractions were dried with N_2_ gas and redissolved in DMSO for *in vitro* biologic studies.

### PAH and quinone analyses.

PAH content in each fraction was determined by an HPLC-fluorescence method that detects a signature group of 16 PAHs (Li et al. 2003). Quinone content was analyzed as described by Cho et al. (2004). Briefly, quinones in the samples were derivatized and evaporated to approximately 50 μL under nitrogen; then, 100 mg zinc, anhydrous tetrahydrofuran, and 200 μL acetic anhydride were added to samples. After heating at 80°C for 15 min, samples were cooled to room temperature and an additional 100 mg zinc was added, followed by an additional 15 min of heating. The reaction was quenched with 0.5 mL water and 2 mL pentane. After centrifugation at 750 × *g* for 10 min, the pentane layer was evaporated to dry and the residue was reconstituted in 50–100 μL dry acetonitrile. 1,2-Naphthoquinone (NQ), 1,4-NQ, phenanthraquinone (PQ), and anthraquinone (AQ) were analyzed by the electron-impact gas chromatography/mass spectrometry technique using an HP MSD mass spectrometer (Hewlitt Packard, Palo Alto, CA) equipped with an automatic sampler (Cho et al. 2004).

### Cell culture and stimulation.

RAW 264.7 cells were cultured in a 5% carbon dioxide in Dulbecco modified Eagle medium (DMEM) containing 10% fetal calf serum, 5,000 U/mL penicillin, 500 μg/mL streptomycin, and 2 mM l-glutamine. For exposure to DEP extracts and its fractions, aliquots of 3 × 10^6^ cells were cultured in six-well plates in 3 mL medium at 37°C for the indicated time periods.

### Cellular staining with fluorescent probes and flow cytometry.

Cells were stained with fluorescent dyes diluted in DMEM, except for annexin V and PI, which were prepared in a commercial binding buffer (Trevigen). The following dye combinations were added for 15–30 min at 37°C in the dark: *a*) 0.25 μg/mL annexin V plus 47.5 μg/mL PI in 500 μL binding buffer (assessment of apoptosis); *b*) 20 nM DiOC_6_ plus 2 μM HE (assessment of ΔΨm and mostly O_2_^•−^ production, respectively); *c*) 100 nM NAO plus 2 μM HE (to assess cardiolipin mass and O_2_^•−^ production, respectively). Flow cytometry was performed using a FACScan (Becton Dickinson, Mountain View, CA) equipped with a single 488-nm argon laser. DiOC_6_, NAO, and annexin V-FITC were analyzed using excitation and emission settings of 488 nm and 535 nm (Fl-1 channel); PI, 488 nm and 575 nm (Fl-2 channel); and HE, 518 nm and 605 nm (Fl-3 channel). Forward and side scatter were used to gate out cellular fragments.

### Preparation of mouse liver mitochondria and experimental conditions.

We removed livers from Balb/c mice and isolated mito-chondria by a standard differential centrifugation procedure as previously described (Xia et al. 2002). Briefly, liver tissue was homogenized with four strokes of a Teflon pestle in buffer A (250 mM sucrose, 1 mM EGTA, and 5 mM HEPES, pH 7.4) on ice. After centrifugation at 1,000 × *g* for 10 min at 4°C, the supernatant was removed and recentrifuged at 10,000 × *g* for 10 min. The pellet was sequentially washed with buffer A and buffer B (buffer A without EGTA). The pellet was resuspended in buffer B and used within 5 hr after isolation. Mitochondrial protein content was determined by the Bradford method (Xia et al. 2002).

Most of the isolated mitochondrial experiments were conducted in a fiberoptic spectrofluorimeter (Ocean Optics, Dunedin, FL), which uses a closed, continuously stirred cuvette at room temperature (Korge et al. 2002). Mitochondria were added to the cuvette at 0.1 mg/mL in a standard buffer containing 250 mM sucrose and 5 mM HEPES, pH 7.4. Substrates, Ca^2+^, PI, inhibitors, and fluorescent indicators were added at the indicated concentrations as shown for each experiment.

### Mitochondrial swelling assay.

Mitochondria (0.1 mg/mL) were incubated in swelling buffer containing 250 mM sucrose, 5 mM HEPES (pH 7.4), 2 μM rotenone, 1 mM PI, and 4.2 mM succinate at room temperature. Mitochondria were then exposed to different chemicals.

Changes in mitochondrial volume were estimated by measuring 90° light scatter with excitation and emission wavelengths set at 520 nm (Walter et al. 2000). Changes in matrix volume were reported as a percentage of maximum (100%) swelling induced by 10μg Ala at the end of each run.

### Measurement of ΔΨm.

TMRM was included at 400 nM, and ΔΨm was estimated at a wavelength of 570 nm (Korge et al. 2002). Decrease in ΔΨm was expressed as percentage decrease in TMRM fluorescence compared with the effect of 1 μM CCCP (100%) in fully energized mitochondria. Light scattering was recorded simultaneously with TMRM fluorescence. In some experiments, ΔΨm was estimated using an ion-selective electrode to measure tetraphenylphosphonium ion (TPP^+^) distribution with a Flex-Ref electrode and Duo 18 recording system (World Precision Instruments, Sarasota, FL). TPP^+^ was added to a final concentration of 3 μM, and the mitochondria were energized by adding succinate at 4.2 mM.

### Calcium Green-5N assay to assess mitochondrial Ca^2+^ retention capacity.

Changes in extramitochondrial Ca^2+^ concentration were followed by measuring Calcium Green-5N (1 μM, salt form) fluorescence at excitation and emission wavelengths of 475 and 530 nm, respectively. Individual Ca^2+^ additions were calibrated by adding known quantities of Ca^2+^ to the buffer in the presence of mitochondria and CCCP to block Ca^2+^ uptake. Addition of chemical materials did not exhibit autofluorescence in our spectrofluorimetry assays.

### Assessment of mitochondrial respiration.

Mitochondrial respiration was carried out in the fiberoptic spectrofluorimeter in the presence of different substrates: succinate, 4.2 mM (complex II); malate/pyruvate/glutamate, 5 mM each (complex I); tetramethyl-*p*-phenylenediamine (TMPD) and ascorbate, 0.2 mM and 2.5 mM, respectively (complex IV) (Korge et al. 2002). The addition of 2 μM CCCP was used as an inducer of maximal respiration. The partial pressure of O_2_ in the buffer was continuously recorded by a fiber-optic oxygen sensor (Foxy Al-300; Ocean Optics, Dunedin, FL).

### Collection of UFPs and assessment of their chemical composition.

UFPs were collected using the Versatile Aerosol Concentration Enrichment System (VACES) in Downey, California, as previously described by Li et al. (2003). Highly concentrated liquid particle suspensions were obtained by connecting the concentrated output flow from the VACES to a liquid impinger (BioSampler; SKC West Inc., Fullerton, CA). Particles were injected into the BioSampler in a swirling flow pattern so that they could be collected in a small volume of water by a combination of inertial and centrifugal forces.

For chemical analysis, we collected two reference filter samples in parallel with the VACES. The first sample was collected on a Teflon filter (47 mm, polytetrafluoroethylene, 2μm pore; Gelman Science, Ann Arbor, MI). Mass concentrations were determined by weighing the Teflon filter before and after each field test in a Mettler 5 Microbalance (Mettler-Toledo Inc., Highstown, NJ). Laboratory and field blanks were used for quality assurance. The Teflon filters were then analyzed by X-ray fluorescence for measurement of trace-element and metal concentrations. The second collection was done on two 47-mm quartz filters (Pallflex Corp., Putnam, CT). These filters were used for measurement of inorganic ions as well as for determining PAH, elemental carbon (EC), and organic carbon (OC) concentrations. A slice of approximately 0.2 cm^2^ from each filter was placed in a platinum boat containing manganese dioxide. The sample was acidified with an aliquot of HCl and heated to 115°C to form CO_2_ as an index of particle-associated carbon. The boat was then inserted into a dual-zone furnace, where MnO_2_ oxidized OC at 550°C and EC at 850°C. A flame ionization detector converted the CO_2_ combustion product to CH_4_ for detection. The remaining filter was divided in two equal parts: one half was analyzed by means of ion chromatography to determine the concentrations of particulate sulfate and nitrate; the other half was analyzed by a HPLC-fluorescence method for detection of a group of signature PAHs as previously described (Li et al. 2003).

### Statistics.

The experiments were reproduced four times, except where otherwise stated. Results were analyzed by Student’s *t*-test, and changes were considered significant at *p* < 0.05.

## Results

### Differential toxicity and mitochondrial effects exerted by aliphatic, aromatic, and polar DEP fractions.

Previous data from our laboratory showed that crude organic DEP extracts mimic the effects of intact particles in ROS production and cytotoxicity (Hiura et al. 1999). Mitochondria play a key role in DEP-induced toxicity, as shown by an early decrease in ΔΨm, loss of inner membrane mass, caspase 9 activation, and onset of apoptosis (Hiura et al. 2000). To clarify which organic chemicals play a role in this cytotoxicity, the crude extract was fractionated by silica gel chromatography, as previously described (Li et al. 2000). Elution with increasingly polar solvents resulted in the recovery of aliphatic, aromatic, and polar fractions in the amounts shown in Table 1. Although the aromatic fraction was enriched for PAHs (Table 2), the polar fraction was devoid of this chemical group but contained an abundance of quinones (Table 3). No quinones were present in the aromatic fraction (Table 3).

**Figure 1:**
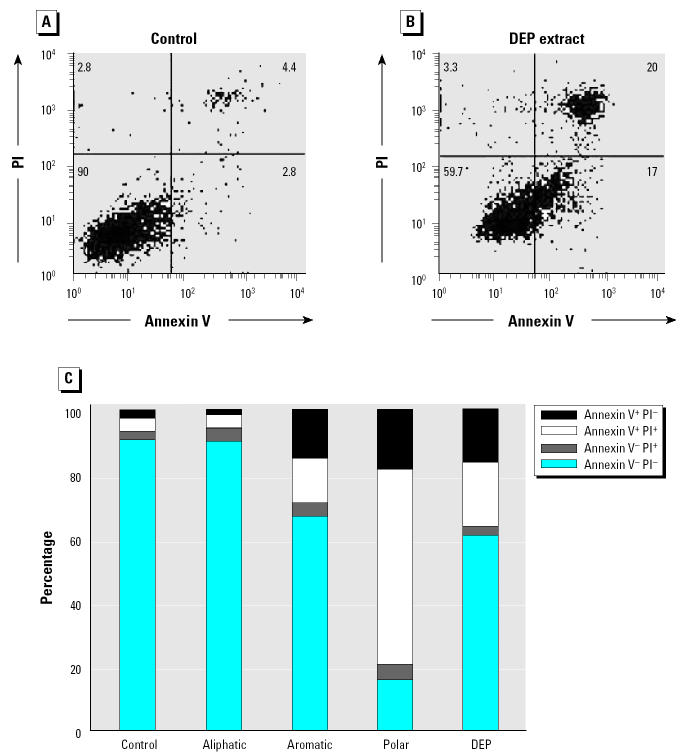
Flow cytometry showing that DEP fractions induce apoptosis in RAW 264.7 cells. (*A*) Control. (*B*) DEP. Cells were treated with 25 μg/mL of the crude DEP extract for 12 hr, stained with annexin V-FITC and PI, and analyzed by flow cytometry. (*C*) Flow data expressed as a stack diagram, in which the crude extract data are compared with the effects of aliphatic, aromatic, and polar fraction, each used at 25 μg/mL; the data are representative of three experiments in which the induction of apoptosis by the crude DEP material, as well as the aromatic and polar fractions, was statistically significant (*p* < 0.05).

RAW 264.7 cells were treated with these chemicals and assessed for evidence of apoptosis (Figure 1). Figure 1A and 1B show representative flow cytometry panels of an experiment that was performed in triplicate. The results demonstrate the induction of annexin V^+^/PI^−^ (lower right) and annexin V^+^/PI^+^ (upper right) cells by the crude extract. These represent early and late apoptotic events, respectively, and can be combined with live (annexin V^−^/PI^−^, lower left) and dead (annexin V^−^/PI^+^, upper left) cells to provide a graphic display of cellular viability/toxicity (Figure 1C). This presentation format demonstrates that the polar fraction is considerably more toxic than the aromatic fraction, whereas the aliphatic fraction has no effect on cell viability (Figure 1C).

**Figure 2:**
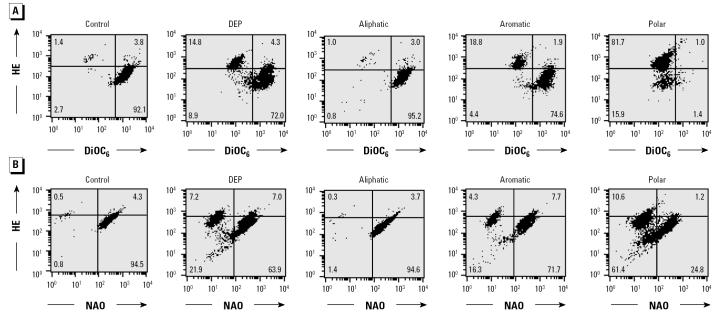
Changes in ΔΨm, mitochondria mass, and ROS production induced by DEP chemicals in RAW 264.7 cells dual-color stained with either (*A*) HE (detects mostly O_2_^•−^) plus DiOC_6_ (ΔΨm) or (*B*) NAO (mitochondria mass) plus HE. RAW 264.7 cells were treated with 100 μg/mL DEP extract or its fractions for 5.5 hr before staining. Data are representative of two experiments.

To explore mitochondrial perturbation, we assessed ΔΨm and ROS production by dual-color DiOC_6_/HE fluorescence (Hiura et al. 1999). DiOC_6_ reflects ΔΨm, whereas HE measures mostly O_2_^•−^ production. This analysis shows that although the aliphatic fraction was inactive, the aromatic and polar fractions induced the appearance of DiOC_6_^low^/HE^high^ subpopulations (Figure 2A). These effects were dose dependent (not shown), with the polar being more active than the aromatic fraction at comparable dose levels (Figure 2). To test whether O_2_^•−^ production is related to inner membrane damage, we also performed dual-color NAO/HE fluorescence (Figure 2B). NAO binds to the inner membrane phospholipid, cardiolipin. Although NAO fluorescence is ΔΨm sensitive, a decrease in fluorescence reflects inner membrane damage. Both polar and aromatic compounds led to a decrease in inner membrane mass, whereas the aliphatic fraction was inactive (Figure 2). Cells with damaged mitochondria also showed increased HE fluorescence, which is in accordance with increased O_2_^•−^ production by cells with reduced ΔΨm (Figure 2A). Overall, the polar fraction was more active than the aromatic fraction in its ability to induce these mitochondrial effects (Figure 2). Taken together, these results demonstrate that the aliphatic, aromatic, and polar fractions exert differential toxic effects on mitochondria and cellular viability.

### Differential effects of the polar fraction on membrane depolarization and PTP opening.

**Figure 3:**
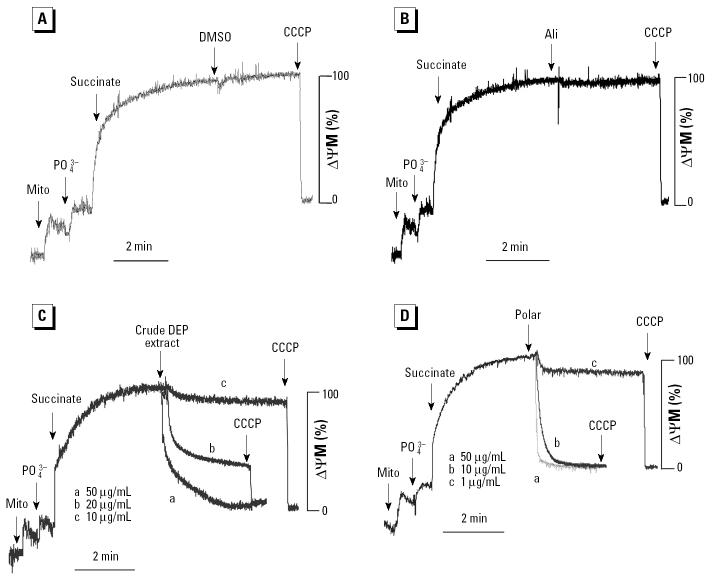
Effects of organic DEP chemicals on ΔΨm in isolated mitochondria (Mito) incubated with 3 μM TPP^+^, 1 mM phosphate (PO_4_^3−^), 4.2 mM succinate, and chemicals. (*A*) DMSO carrier. (*B*) Aliphatic fraction at 100 μg/mL. (*C*) Crude DEP extract. (*D*) Polar fraction. DEP extract and polar fraction were added as indicated by the arrows; CCCP was used to completely depolarize the mitochondria and to serve as a quantitative control. Data are representative of four experiments.

To further explore the action of functionalized DEP chemical groups on mitochondrial function, we performed a series of studies in isolated liver mitochondria. First, ΔΨm was recorded with a TPP^+^ electrode after the addition of phosphate and succinate to the mitochondrial preparation (Kushnareva and Sokolove 2000). The addition of CCCP, a protonophore uncoupler, led to a quick dissipation of the ΔΨm (Figure 3A). Although the carrier (DMSO) and the aliphatic fraction were inactive (Figure 3A,B), the crude extract as well as the polar fraction induced a dose-dependent decline in ΔΨm (Figure 3C,D). The polar material was more potent and induced a faster rate of depolarization (Figure 3D).

**Figure 4:**
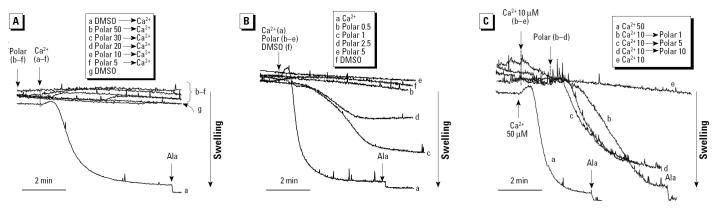
Effects of DEP and the polar fraction on mitochondrial swelling. (*A*) 50 μM Ca^2+^ added after DMSO and different doses of polar fraction (5, 10, 20, 30, 50 μg/mL); the control was DMSO alone. The data are representative of four experiments in which the inhibitory effect of polar concentrations ≥5 μg/mL on Ca^2+^-induced swelling was statistically significant (*p* < 0.01). (*B*) 50 μM Ca^2+^ introduced to induce swelling as a positive control; polar material (0.5, 1, 2.5, 5 μg/mL) was added in the absence of a Ca^2+^ stimulus, and the control was DMSO alone. See “Materials and Methods” for details. (*C*) When previously loaded with a small amount of 10 μM Ca^2+^, the subsequent addition of the polar material (1, 5, 10 μg/mL) induced near-maximal mitochondrial swelling at all doses tested.

If mitochondria are well polarized, addition of a large Ca^2+^ load leads to matrix Ca^2+^ uptake and PTP opening (Korge et al. 2002). PTP opening leads to mitochondrial swelling, which can be followed by using 90° light scatter in a spectrophotometer (Figure 4A, a). In mitochondria that had not been subjected to a Ca^2+^ load, addition of a small and nondepolarizing polar dose (1–2.5 μg/mL; Figure 3) caused spontaneous induction of mitochondrial swelling (Figure 4B, c and d). Compared with the lack of response to the DMSO carrier, these results were statistically significant (*p* < 0.01). In contrast, higher doses of the polar fraction (≥5 μg/mL) caused a statistically significant (*p* < 0.01) inhibition of Ca^2+^-induced mitochondrial swelling (Figure 4A). The same effect (*p* < 0.01) was seen with the crude DEP extract (not shown). This inhibition of swelling can be attributed to the ΔΨm-reducing effects of these higher concentrations. This is similar to the ΔΨm dissipation by CCCP, which prevents the rise in matrix Ca^2+^ required for PTP opening. If, on the other hand, matrix Ca^2+^ is already elevated, ΔΨm depolarization promotes PTP opening because the PTP open probability is voltage dependent and increases with depolarization. To test this theory, isolated mitochondria were preexposed to a small Ca^2+^ load (10 μM) that is insufficient to induce PTP opening, and then exposed to a higher polar concentration range. This led to a dose-dependent induction of mitochondrial swelling at all doses tested (Figure 4C). DMSO and the aliphatic fraction had no effect on mitochondrial swelling (not shown).

**Figure 5:**
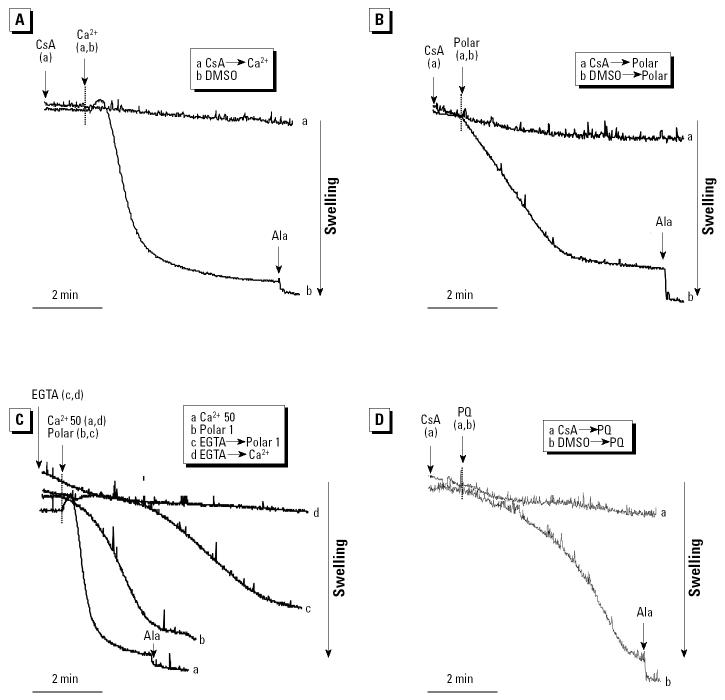
Calcium-dependent PTP transition by the polar fraction and PQ in mitochondria incubated in swelling buffer. Mitochondria were then incubated with 1 μM CsA or DMSO before the addition of 50 μM Ca^2+^ (*A*), 1 μg/mL polar fraction (*B*), and 5 μM PQ (*D*). (*C*) EGTA was added before the introduction of 1 μg/mL polar fraction. See “Materials and Methods” for details. The data are representative of four experiments, in which the swelling effect of the polar fraction and PQ where both statistically significant at *p* < 0.01. The inhibition by CsA was also statistically significant at *p* < 0.01.

To confirm that mitochondrial swelling induced by the crude extract and polar fraction was due to PTP opening, we examined the effects of the PTP inhibitor CsA (Figure 5). Similar to its effect on Ca^2+^-induced swelling, CsA added before the addition of the polar fraction (Figure 5A, a) abrogated polar-induced mitochondrial swelling in a statistically significant fashion (*p* < 0.01) (Figure 5B). Ca^2+^-dependent mitochondrial swelling by the polar fraction was confirmed by prior addition of EGTA, which led to a significant reduction in the rate and magnitude of mitochondrial swelling in the presence of 1 μg/mL of the polar material (Figure 5C, b vs. c).

The polar fraction contains a number of chemicals, among which the quinones participate in the generation of oxidative stress and covalent protein modification (Penning et al. 1999). We tested a number of DEP quinones (Table 3) for their effects on mitochondrial swelling, including PQ, 1,2-naphthaquinone, and AQ. PQ induced statistically significant (*p* < 0.01) mitochondrial swelling with slower kinetics than did the Ca^2+^ load stimulus (Figure 5D). This effect was totally suppressed by CsA, indicating that quinones stimulate PTP activity in a Ca^2+^-dependent fashion (Figure 5D). Similar results were obtained with 1,2-naphthaquinone, whereas a nonredox-cycling quinone, AQ, was inactive (not shown). These results suggest that redox-cycling quinones play a role in the cytotoxic effects of DEPs on the mitochondrion.

All considered, the data presented indicate that polar chemicals induce mitochondrial swelling due to PTP opening. This involves direct action on the PTP at low doses, as well as rapid-onset ΔΨm depolarization at higher doses, provided that the matrix Ca^2+^ concentration is already elevated. In the absence of Ca^2+^ loading, higher polar doses inhibit mitochondrial swelling, most likely due to interference in Ca^2+^ accumulation as a result of ΔΨm depolarization.

### Interference in the function of respiratory complexes by the polar fraction.

**Figure 6:**
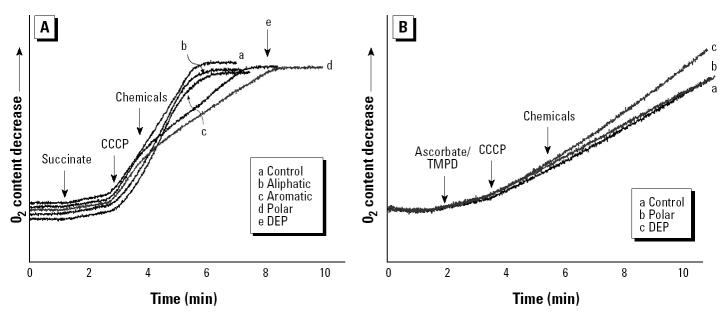
Effects of organic DEP chemicals on mitochondrial respiration. (*A*) Succinate as a complex II substrate. (*B*) Ascorbic acid/TMPD as complex IV substrates. See “Materials and Methods” for details. Maximal mitochondrial respiration was initiated by 2 μM CCCP before the addition of DEP or its fractions at 50 μg/mL. Data are representative of three experiments.

Mitochondrial uncoupling increases mitochondrial respiration, which can be assessed by measuring oxygen consumption with an oxygen-sensing electrode (Figure 6). Although the polar fraction increased mitochondrial respiration as a consequence of its depolarizing effect (not shown), the induction of maximal respiration by CCCP in the presence of succinate showed that subsequent addition of the polar fraction caused a slowing of respiration (Figure 6A). The crude DEP extract had the same effect, whereas the aromatic or aliphatic fractions did not affect maximal mitochondrial respiration (Figure 6A). These findings indicate that the polar fraction and crude DEPs interfere in the function of complex II in the inner membrane. Similar results were obtained when using malate/glutamate/pyruvate, which are substrates for complex I (not shown). However, there was no effect when ascorbate and TMPD were used, implying that complex IV was not affected by the polar chemicals (Figure 6B). We propose that exogenous quinones present in the polar fraction might compete with the ubiquinones, which play a critical role in electron transfer in the inner membrane complexes. Transfer of those electrons to molecular dioxygen could explain O_2_^•−^ production.

### Unique effects on ΔΨm, mitochondrial swelling, and Ca^2+^ retention capacity exerted by the aromatic fraction and PAHs.

**Figure 7:**
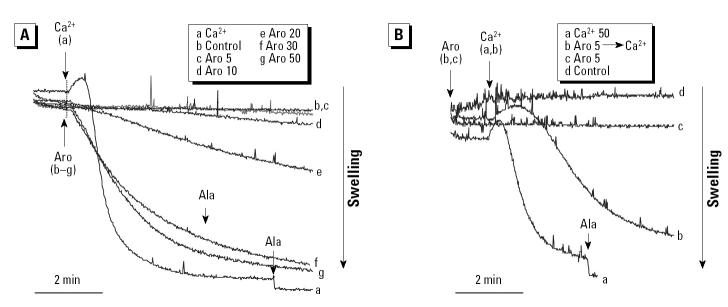
Effects of the aromatic fraction on mitochondrial swelling. (*A*) 50 μM Ca^2+^, DMSO alone, or different doses of aromatic (Aro) fraction (5, 10, 20, 30, 50 μg/mL); mitochondrial swelling was statistically significant (*p* < 0.01) at aromatic doses ≥20 μg/mL. (*B*) 50 μM Ca^2+^, 5 μg/mL aromatic fraction (Aro) followed by 50μM Ca^2+^, 5 μg/ml Aro alone, or control (DMSO alone). The data are representative of four experiments.

Treatment with the aromatic fraction induced a dose-dependent ΔΨm decrease in isolated liver mitochondria at doses > 10 μg/mL (not shown). Unlike that observed with the polar fraction (Figure 3D), this depolarization was incomplete compared with CCCP (not shown). In addition, the aromatic fraction induced spontaneous mitochondrial swelling in a dose-dependent fashion (Figure 7A, b–f). In non-Ca^2+^-loaded mitochondria, this effect started at aromatic doses ≥10 μg/mL (Figure 7A), whereas lower doses (e.g., 5 μg/mL) actually inhibited Ca^2+^-induced swelling (Figure 7B). This is the opposite from the effect observed with the polar fraction, which interfered in mitochondrial swelling at high doses but induced spontaneous swelling at low doses (Figure 4B,C). Taken together, these data suggest that differences in the chemical composition of the aromatic and polar fractions lead to differential effects on mitochondrial function.

**Figure 8:**
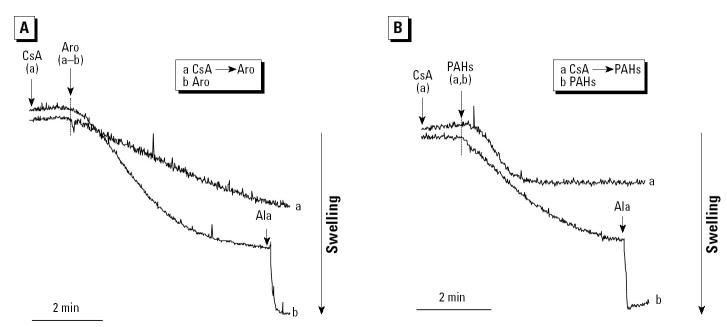
Effects of the aromatic fraction and PAHs on mitochondrial swelling. (*A*) 1 μM CsA followed by the addition of 20 μg/mL aromatic fraction (Aro) or 20 μg/mL Aro alone; the experiment was reproduced four times, with statistically significant (*p* < 0.05) inhibition of mitochondrial swelling by CsA. (*B*) CsA followed by 7.8 μg/mL PAHs or PAHs alone; the experiment was reproduced four times, with statistically significant stimulation by PAHs (*p* < 0.01) and inhibition (*p* < 0.01) of the swelling effect by CsA. See “Materials and Methods” for details.

PAHs are the main components of the aromatic fraction and are capable of inducing apoptosis (Li et al. 2000). To test if PAHs exert an effect on isolated mitochondria, we used a commercial source composed of 16 DEP PAHs to conduct the swelling assay. This demonstrated that the PAH mix can induce slow-onset swelling in non-Ca^2+^-loaded mitochondria, which mimics the effects of the aromatic fraction (Figure 8). This swelling effect was incomplete and was partially but statistically significantly (*p* < 0.05) inhibited by CsA (Figure 8B). CsA exerted the same effect on the induction of swelling by the aromatic fraction (Figure 8A).

### Use of mitochondrial calcium retention capacity to study differences between the polar and aromatic fractions on PTP opening.

**Figure 9:**
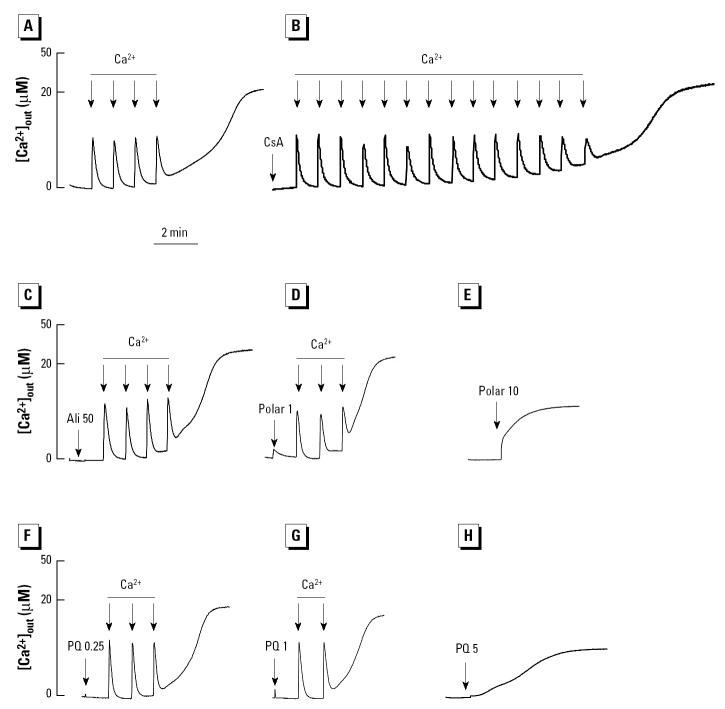
Effect of the polar fraction and quinones on the Ca^2+^ retention capacity of isolated mitochondria incubated with 1 μM Calcium Green-5N. After the addition of mitochondria, the following chemicals were added: (*A*) DMSO (carrier), (*B*) CsA, (*C*) aliphatic (Ali), (*D*) 1 μg/mL polar fraction, (*E*) 10 μg/mL polar fraction, (*F*) 0.25 μM PQ, (*G*) 1 μM PQ, and (*H*) 5 μM PQ. Each arrow represents one 5 μM Ca^2+^ pulse. Data are representative of four experiments.

Calcium Green-5N is a fluorescent dye that can be used to assess the Ca^2+^ retention capacity of isolated mitochondria. The addition of small amounts of Ca^2+^ leads to a rapid matrix uptake into isolated energized mitochondria (Figure 9A). With repeated Ca^2+^ pulses, matrix Ca^2+^ eventually triggers PTP opening, which leads to depolarization and release of Ca^2+^ from the matrix (Figure 9A). This leads to a precipitous and sustained increase in fluorescence intensity (Figure 9A). This response is statistically significantly (*p* < 0.01) inhibited by CsA, which increased the number of Ca^2+^ pulses from 4 to 14 (Figure 9B). The aliphatic fraction had no effect on the number of Ca^2+^ pulses (Figure 9C), whereas 1 μg/mL of the polar material reduced the number of Ca^2+^ pulses required to trigger PTP transition (Figure 9D). This finding is compatible with the ability of the polar fraction to induce spontaneous mitochondrial swelling in a Ca^2+^-dependent fashion (Figure 4C). Higher polar concentrations induced immediate release of ambient accumulated Ca^2+^, which reflects its depolarizing effect (Figure 9C). Similar results were obtained with the crude DEP extract: a reduction in the required number of Ca^2+^ pulses at low doses and precipitous Ca^2+^ release at high doses (not shown).

Because we have shown that DEP quinones mimic the effect of the polar fraction in spontaneous mitochondrial swelling, we also tested these quinones in the Calcium Green-5N assay. PQ reduced the required number of Ca^2+^ applications to achieve PTP from 3, to 2, to 0 at PQ concentrations of 0.25, 1, and 5 μg/mL, respectively (Figure 9F–H). CsA could significantly (*p* < 0.01) increase the number of Ca^2+^ pulses required for precipitous Ca^2+^ release in the presence of PQ, suggesting PTP involvement. Similar results were obtained with 1,2-NQ but not with AQ (not shown).

**Figure 10:**
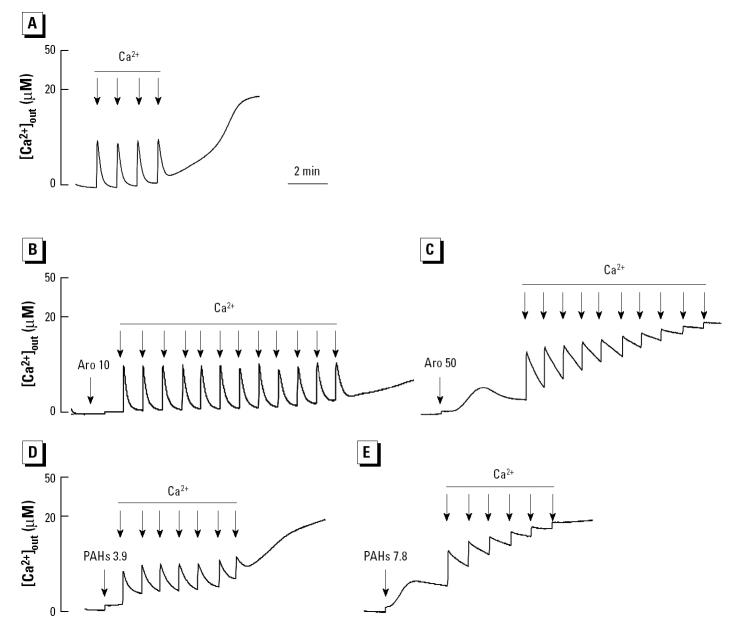
Effect of the aromatic fraction and PAHs on the Ca^2+^ retention capacity of isolated mitochondria incubated with 1 μM Calcium Green-5N. After the addition of mitochondria, the following chemicals were added: (*A*) DMSO, (*B*) aromatic (Aro) 10 μg/mL, (*C*) Aro 50 μg/mL, (*D*) PAH mix 3.9 μg/mL, and (*E*) PAH mix 7.8 μg/mL. Each arrow represents one 5 μM Ca^2+^ pulse. Data are representative of three experiments.

Examination of the aromatic fraction in the Calcium Green-5N assay showed that doses < 10 μg/mL increased the Ca^2+^ retention capacity (Figure 10A,B). This is in keeping with the ability of the aromatic fraction to inhibit Ca^2+^-induced PTP opening in this dose range (Figure 7B). At higher doses, the aromatic fraction induced a short Ca^2+^ burst, probably related to ΔΨm depolarization, which is followed by a progressive decline in the ability of the matrix to accumulate Ca^2+^ (Figure 10C). This depolarization was incomplete and not CsA sensitive (not shown). In order to determine whether this effect is related to the PAHs present in the aromatic fraction, the DEP PAH mixture was separately tested. PAHs mimicked the effect of the aromatic fraction in the low and high dose range (Figure 10D,E). Taken together, these results confirm that the polar and aromatic DEP compounds exert fundamentally different actions on mitochondria.

### Effects of ambient UFPs on mitochondrial responses.

**Figure 11:**
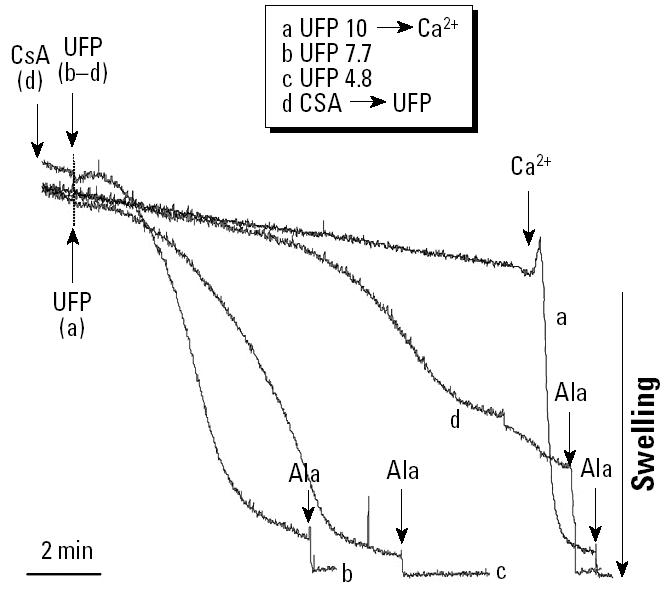
Effects of UFP on mitochondrial swelling conducted in the presence of 10 μg/mL UFP followed by Ca^2+^ (50 μM), 7.7 μg/mL UFP without Ca^2+^ loading, 4.8 μg/mL UFP without Ca^2+^ loading, or 1 μM CsA followed by 7.7 μg/mL UFP. Data are representative of three experiments.

A key question is whether the effects of the DEP chemicals can be reproduced with intact DEP and “real-life” ambient particles (Li et al. 2003). Intact DEPs induce apoptosis (Hiura et al. 1999), and ambient UFPs induce structural damage and lodge inside mitochondria in RAW 264.7 cells and epithelial cells (Li et al. 2003). When UFPs, collected by a particle concentrator in the Los Angeles Basin (Kim et al. 2001), were tested in the mitochondrial swelling assay, we observed spontaneous PTP opening at doses of 4.8 and 7.7 μg/mL in non-Ca^2+^-loaded mitochondria (Figure 11, b and c). Swelling was partially reversed by CsA (Figure 11, d). At a dose of 1.9 μg/mL, UFPs did not induce spontaneous PTP opening but interfered with Ca^2+^-induced swelling (not shown). This is similar to the effect of sonicated DEP, which interfered in Ca^2+^-induced mitochondrial swelling in a dose-dependent fashion but failed to induce spontaneous swelling (Table 4). This could relate to differences in the particle size (the DEP powder used here contains particle aggregates) as well as differences in the bioavailability of surface chemical compounds on these particles. The chemical composition of UFPs is shown in Table 5. In contrast to the particulate pollutants, artificial polystyrene microspheres (size < 100 nm) did not exert an effect on mitochondrial swelling, and the mitochondria remained fully responsive to Ala (Figure 11, a).

**Figure 12:**
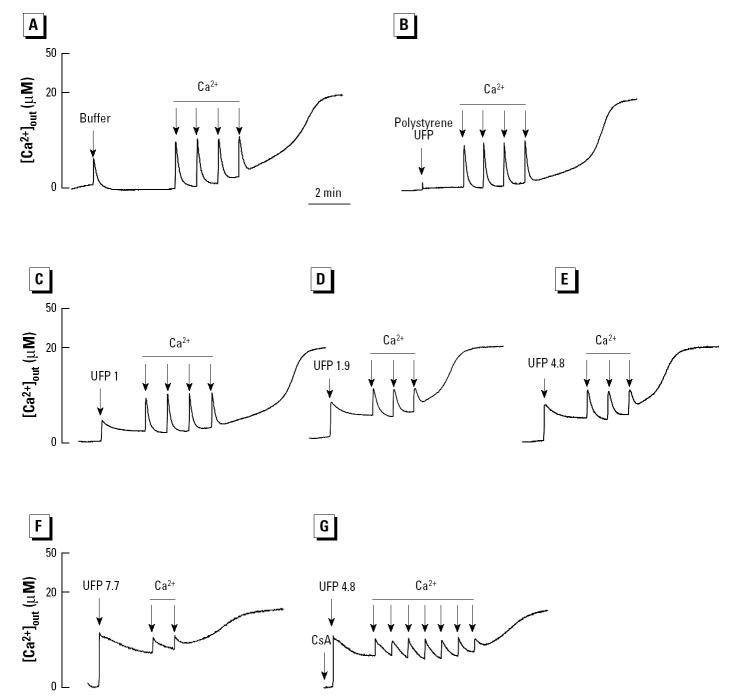
Effect of UFPs on Ca^2+^ retention capacity of isolated mitochondria incubated with 1 μM Calcium Green-5N. After the addition of mitochondria, the following chemicals were added: (*A*) carrier buffer, (*B*) 10 μg/mL polystyrene microspheres, (*C*) 1 μg/mL UFP, (*D*) 1.9 μg/mL UFP, (*E*) 4.8 μg/mL UFP, (*F*) 7.7 μg/mL UFP, (*G*) CsA followed by the addition of 4.8 μg/mL UFP. Each arrow represents one 5 μM Ca^2+^ pulse. Data are representative of three experiments.

In the Calcium Green-5N assay, ambient UFPs induced instantaneous Ca^2+^ release but reduced Ca^2+^ retention capacity in a dose-dependent manner (Figure 12A vs. Figure 12C–F). CsA prevented this effect (Figure 12G). Sonicated DEPs had a similar effect that was also CsA sensitive (Table 4). In contrast, polystyrene microspheres (80 nm) had no effect on Ca^2+^ retention capacity (Figure 12B). This suggests that the effect of the ambient UFP is dependent on their content of redox-cycling chemicals. Taken together with the data shown in Figure 11, the UFP effects appear to be a summation of the effects of polar and aromatic chemical compounds.

## Discussion

In this study we looked at the effects of distinct DEP chemical fractions on mitochondrial function. A major effect of the polar fraction was to promote mitochondrial swelling, both directly at the level of PTP opening and indirectly by promoting ΔΨm depolarization. Mitochondrial swelling by the polar fraction and the redox-cycling quinones involved the induction of Ca^2+^-dependent PTP opening, as determined by the inhibitory effect of CsA and EGTA. Polar interference in inner membrane function likely targets membrane complexes I–III, as determined using different substrates in the mitochondrial respiratory chain. The polar fraction also contains chemical substances that induce mitochondrial swelling, even at low doses that have no effect on ΔΨm. This effect could be mimicked by DEP quinones, which are enriched in the polar fraction. Although the aliphatic fraction failed to affect mitochondrial function, the aromatic fraction induced a decrease in ΔΨm that is likely secondary to PTP perturbation. This effect is mostly Ca^2+^ independent and can be mimicked by PAHs. At low doses, the aromatic fraction increased the Ca^2+^ retention capacity, suggesting interference in PTP function. However, at higher doses, the aromatic fraction induced partial ΔΨm depolarization, which could promote swelling if matrix Ca^2+^ was already elevated. The polar and aromatic effects on isolated mitochondria could be mimicked, in part, by ambient UFPs and intact DEPs, which contain an abundance of both functionalized chemical species. In contrast, commercial polystyrene nanoparticles, which lack these chemicals, were inactive. The above effects on isolated mitochondria were accompanied by effects on apoptosis and ΔΨm in intact RAW 264.7 cells.

There is a paucity of data about the mechanisms by which ambient PM induces adverse health effects. There is also a considerable debate as to whether the particles themselves or their chemical components are responsible for injurious effects in the respiratory tract and cardiovascular system (Brown et al. 2000; Oberdörster 1996). Our view is that both the particles and the chemicals are important. First, the particles are effective carriers of chemical compounds, many of which are semi-volatile organic substances that will not otherwise gain access to the deeper regions of the lung. Second, the particle surface may act as an important catalyst for chemical reactions involved in ROS generation (Brown et al. 2000). Third, particles localize inside target cells, and it is possible that their subcellular localization may be determined by chemical composition. This could explain why ambient UFPs lodge inside mitochondria in epithelial cells and macrophages and why these particles are more potent than larger-sized particles in perturbing mitochondrial function (Figure 12). One possibility is that the negative charge of the mitochondrial matrix or the positive charge in the intermembrane space attracts chemical dipoles that are present in the polar material. Another possibility is that the large surface area of UFPs may promote the bioavailability of the absorbed chemicals. UFPs are known to have increased solubility, compared with larger sized particles of the same composition because of the increased surface-to-volume ratio for smaller particle sizes (Navrotsky 2001). This could explain why UFPs induce spontaneous swelling, whereas the major effect of DEPs is inhibition of Ca^2+^-induced swelling (Table 4). PAHs and quinones are representative chemical groups that may be released in different amounts from DEPs and UFPs. The type of PAH (e.g., 4-, 5-, or 6-ring PAHs) could also play a role in determining bioavailability.

How does mitochondrial perturbation lead to adverse PM health effects? An obvious mechanism is ROS production in mitochondria (Hiura et al. 1999). Although oxidative stress is increasingly recognized as a key component in tissue damage by DEPs, there is still a great deal of uncertainty about the origin of ROS. It is possible that one-electron transfers to molecular dioxygen in the mitochondrial inner membrane could contribute to O_2_^•−^ generation. This effect is compatible with the effects of the polar fraction on inner membrane complexes I–III (Figure 6) and increased HE fluorescence in RAW 264.7 cells (Figure 2). We propose that quinones play a role in redirecting electron transfer to molecular O_2_ in the inner membrane. This effect could be enhanced by PTP transition, which disrupts the ΔΨm and increases O_2_^•−^ generation (Zoratti and Szabo 1995). This does not imply that O_2_^•−^ generation by mitochondria is the only PM-induced source of ROS production. In fact, it is well known that in phagocytic cells mitochondria are a minor source for ROS production compared with NADPH oxidase and lysosomes (Bassoe et al. 2003).

PM contains a number of polar chemical substances, including quinones, ketones, aldehydes, sulfur compounds, and dibutyl phthalate (Shuetzle et al. 1981). Although much needs to be learned about the biologic effects of these substances, there is a substantive biologic literature describing tissue injury by quinones (Penning et al. 1999). The endogenous ubiquinones play a key role in one-electron transfers in the mitochondrial inner membrane as well as PTP transition (Fontaine et al. 1998; Walter et al. 2000). Walter et al. (2000) described three classes of ubiquinones that affect the PTP: group I ubiquinones (Ub0, decyl-Ub, Ub10, 2,3,5-trimethyl-6-geranyl-1,4-benzoquinone, and 2,3-dimethyl-6-decyl-1,4-benzoquinone) act as PTP inhibitory quinones that enhance the Ca^2+^ load required for PTP opening; group II quinones [2,3-dimethoxy-5-methyl-6-(10-hydroxydecyl)-1,4-benzoquinone and 2,5-dihydroxy-6-undecyl-1,4-benzoquinone] act as PTP-activating quinones that dramatically decrease the Ca^2+^ load required for PTP opening; group III or PTP-inactive quinones [2,3,5-trimethyl-6-(3-hydroxyisoamyl)-1,4-benzoquinone and Ub5] are neutral in their effect but have the ability to counteract the effects of group I and II quinones (Walter et al. 2000). Although the mechanism of PTP perturbation is unclear, it has been proposed that competition between these groups is mediated through the occupancy of a common quinone binding site in the PTP (Walter et al. 2000). According to this hypothesis, ligation by stimulating (group II) quinones facilitates PTP opening at a relatively small Ca^2+^ load, whereas a larger Ca^2+^ load would be required to access the Ca^2+^ binding site when liganded with inactive (group III) quinones, and an even larger Ca^2+^ load when liganded with inhibitory (group I) quinones (Walter et al. 2000). If a mixture of quinones is present, they could compete in a concentration- and affinity-dependent manner for binding to the PTP site.

Although the applicability of this model to exogenous quinones is uncertain, it is interesting that redox-cycling NQs have been shown to induce Ca^2+^-dependent, CsA-sensitive PTP transition (Henry and Wallace 1995; Palmeira and Wallace 1997). On the other hand, non–redox-cycling quinones with sulfhydrylarylating potential (e.g., benzoquinone) induce direct, Ca^2+^-independent depolarization and mitochondrial swelling that is insensitive to CsA inhibition (Henry and Wallace 1995; Palmeira and Wallace 1997). These findings are compatible with our data that redox-cycling DEP quinones (e.g., PQ and 1,2-NQ) induce a Ca^2+^-dependent, CsA-sensitive PTP transition, whereas a non–redox-cycling DEP quinone (AQ) had no effect (Figure 5D). This suggests that the redox-cycling quinones present in DEPs are responsible for PTP transition. In the absence of Ca^2+^ loading, this effect disappears at higher polar concentrations that prevent Ca^2+^ accumulation (Figure 4C, Figure 9D,E). The mechanism by which exogenous quinones perturb PTP activity is unknown. One possibility is binding to the putative ubiquinone binding site mentioned above. Another is the oxidative modification of thiol-dependent PTP components by redox-cycling quinones (Henry and Wallace 1995; Palmeira and Wallace 1997). Whatever the exact explanation, our data indicate that DEP quinones affect mitochondrial function independent of other biologic effects these compounds may have.

It is interesting that the aromatic fraction differs from the polar fraction in its effect on mitochondrial function. The key difference appears to be the ability of the aromatic compounds to interfere in Ca^2+^-induced PTP opening at low doses (Figure 10B) while inducing mostly CsA-insensitive swelling at higher doses (Figure 7A). These effects are mimicked by the PAHs, suggesting that they play a key role in the toxic effect of the aromatic compounds (Figure 10D,E). Although we lack a definitive molecular explanation for the PAH effects, their action at lower doses resembles PTP inhibition by CsA (Figure 10D). Whether this represents occupation of an inhibitory binding site similar to group II ubiquinones or interference in cyclophylin D binding to the pore is unknown. Lemasters and colleagues have postulated that the PTP has two open conductance modes: one activated by Ca^2+^ and inhibited by CsA and the other independent of Ca^2+^ and CsA insensitive (He and Lemasters 2002; Lemasters et al. 2002). Induction of the Ca^2+^-independent open state has been suggested to be mediated by oxidative chemicals, such as phenylarsine oxide (PAO) and HgCl_2_, which lead to misfolding of integral membrane proteins at high doses (He and Lemasters 2002). It is possible that high doses of aromatic chemicals could act in similar fashion (Lemasters et al. 2002). According to the protein misfolding hypothesis, cyclophilin D protects against this effect by acting as a chaperone for the damaged proteins (Lemasters et al. 2002). That could lead to decreased cyclophilin D binding to the PTP, which may explain why the aromatic fraction interferes in Ca^2+^-induced PTP opening (Figure 7B). At a high aromatic dose, the number of misfolded protein clusters could overwhelm the ability of the chaperones to prevent nonspecific channel formation, leading to CsA-insensitive mitochondrial swelling (Figure 7A).

We have frequently referred to the role of Ca^2+^ in PM-induced mitochondrial effects, including the fact that certain quinones affect mitochondrial function and PTP opening in a Ca^2+^-dependent fashion (Henry and Wallace 1995). PAH diol epoxides have been shown to increase cytosolic Ca^2+^ in airway epithelial cells (Jyonouchi et al. 2001), which theoretically could affect mitochondrial function, as demonstrated by the ability of some PAH species to induce apoptosis (Solhaug et al. 2004). In addition to the contribution of chemicals, the particles themselves play a role in intracellular Ca^2+^ release, as demonstrated by treating alveolar macrophages with carbon black particles (Brown et al. 2004).

In addition to using a Ca^2+^-dependent pathway, redox-cycling DEP chemicals may perturb the PTP in a thiol-dependent manner. In this regard, Constantini et al. (1996) proposed that oxidation of vicinol thiol groups in the PTP by ROS or electrophilic chemicals may lead to induction of permeability transition. Bernardi and colleagues have provided data that suggest that two distinct thiol groups are implicated in modulating PTP activity (Chernyak and Bernardi 1996; Constantini et al. 1996). One thiol group is sensitive to glutathione (GSH) oxidation, whereas the other responds to the redox state of the matrix NAD(P). The adenine nucleotide transporter (ANT) protein, a proposed structural PTP component, has three cysteine residues that show differential reactivity toward various thiol and oxidizing reagents in a conformation-dependent fashion (Majima et al. 1993, 1994, 1995). These cysteines could represent the thiol groups that regulate cyclophilin D binding as well as the effects of membrane potential on the PTP. This could explain the synergy between intracellular Ca^2+^ flux and oxidative stress in PTP opening. Interestingly, ANT uses its vicinal thiols to bind to a PAO column (Halestrap et al. 1997). Treatment of isolated mitochondria with a crude DEP extract prevents ANT binding to PAO, suggesting that this protein is oxidatively modified at vicinal thiol groups (Xia et al., unpublished data). The thiol hypothesis also explains the prevention of mitochondrial damage by *N*-acetylcysteine, which, in addition to its effect as a radical scavenger, serves as a precursor for GSH synthesis as well as electrophilic binding to prooxidative DEP chemicals (Xiao et al. 2003). Under physiologic conditions, GSH may play an important role in protecting the vicinal thiols associated with the PTP, hence the association of a drop in GSH levels with DEP-induced apoptosis.

A final point of interest is the potent effect of ambient UFPs on mitochondrial function, compared with no effect from commercial UFPs (Figure 11). This finding is of great importance to the burgeoning field of nanotechnology, which has attracted attention because of the possible interference of nanoparticles in biologic processes (Brumfiel 2003). Although it is possible that very small particles may exert toxic effects and induce intracellular Ca^2+^ flux based on their small size and high surface area, independent of their chemical makeup (Brown et al. 2001, 2004), our data indicate that the injurious effect of ambient UFP is dependent on chemical composition. In addition to the presence of organic chemicals, transition metals may contribute to particle toxicity. By using a mitochondrial end point, we have shown that it is possible to develop a mechanistic approach to particle toxicity. Similar approaches could be used to study the effects of commercial nanoparticles, which, in addition to their chemical composition, may exert mitochondrial effects based on size, surface area, and surface charge.

## Correction

The concentration of DEP extract and its fractions was incorrect in Figure 2 of the manuscript published online; it has been corrected here.

## Figures and Tables

**Table 1:** Recovery of each fraction from 1.2 g DEPs.

Fraction	Elution solvent	Solvent	Amount (mg)	Recovery (%)*^a^*
Aliphatic	Hexane	Hexane	281.4	23.5
Aromatic	Hexane:MC (3:2)*^b^*	MC	125.6	10.5
Polar	MC:methanol (1:1)*^b^*	MC	119.8	10.0
Total			526.8	44.0

MC, methylene chloride.

a From 1.2 g DEPs, 347.6 mg asphaltene was recovered; this represents 29% recovery.

b Vol:vol.

**Table 2:** PAH content in each DEP fraction (ng/1.2 g DEPs).

PAH	Crude extract	Aliphatic	Aromatic	Polar
NAP	10,149	25.5	4,420	0
ACE	7,470	0	513	0
FLU	17,483	0	7,461	0
PHE	179,714	17.2	133,069	0
ANT	2,759	0	1,133	145
FLT	77,278	0	54,122	1,266
PYR	60,425	0	28,024	59.6
BAA	10,349	0	7,392	0
CRY	18,026	0	9,237	0
BBF	5,510	0	2,053	0
BKF	2,275	0.33	391	0
BAP	1,777	0.51	30.2	0
DBA	1,841	0.69	106	0
BGP	2,104	1.32	130	0
IND	2,045	0	119	0

Abbreviations: ACE, acenaphthalene; ANT, anthracene; BAA, benzo(*a*)anthracene; BAP, benzo(*a*)pyrene; BBF, benzo(*b*)fluoranthene; BGP, benzo(*g,h,i*)perylene; BKF, benzo(*k*)fluoranthene; CRY, chrysene; DBA, dibenz(*a,h*)anthracene; FLT, fluoranthene; FLU, fluorene; IND, indeno(1,2,3-*c,d*)pyrene; NAP, naphthalene; PHE, phenanthrene; PYR, pyrene.

**Table 3:** Quinone content in DEP fractions (ng/mg fraction).

Quinone	Crude extract	Aliphatic	Aromatic	Polar
1,2 NQ	22.34	ND	ND	25.09
1,4 NQ	19.94	ND	ND	75.88
9,10 PQ	18.73	ND	ND	66.25
9,10 AQ	69.34	ND	ND	405.02

ND, none detected.

**Table 4:** Comparison of DEP and UFP effects on isolated mitochondria.

Assay	DEP particle	Ambient UFPs
ΔΨm	Dose-dependent delayed or rapid depolarization	Rapid depolarization
	CsA insensitive	CsA insensitive
Mitochondrial Ca^2+^ retention capacity	Decreased retention capacity	Decreased retention capacity
	CsA sensitive	CsA sensitive
Mitochondrial swelling	Dose-dependent inhibition of Ca^2+^-induced swelling	Inhibition of Ca^2+^-induced swelling at low doses (1 μg/mL)
	No spontaneous swelling effects at any dose	Spontaneous swelling at doses > 1.9 μg/mL
		Partially CsA sensitive

All assays were performed as described in “Materials and Methods”; DEPs were sonicated and tested in the dose range 1–50 μg/mL.

**Table 5:** Chemical composition of UFPs (percentage of PM mass).

Major elements (%)	Inorganic ions (%)	EC	OC	PAH
Na (0.84)	Nitrate (4.9)			PHE (1.75)
Al (8.80)	Sulfate (17.6)			FLT (2.72)
Si (14.19)				PYR (2.94)
Cl (0.10)				BAA (1.90)
K (0.67)				CRY (2.53)
Ca (2.05)				BBF (2.39)
Ti (0.47)				BKF (1.04)
V (0.08)				BAP (2.45)
Cr (0.07)				BGP (10.38)
Mn (0.09)				IND (3.04)
Fe (3.20)				
Ni (0.05)				
Cu (0.19)				
Zn (0.10)				
Br (0.01)				
Sr (0.01)				
Zr (0.01)				
Ba (0.10)				
Pb (0.02)				
Total 31%	23%	2%	41%	31.1%

Abbreviations: BAA, benzo(*a*)anthracene; BAP, benzo(*a*)pyrene; BBF, benzo(*b*)fluoranthene; BGP, benzo(*ghi*)perylene; BKF, benzo(*k*)fluoranthene; CRY, chrysene; FLT, fluoranthene; IND, indeno(1,2,3-*cd*)pyrene; PHE, phenanthrene; PYR, pyrene. All species are expressed as a percentage of the total PM mass except PAHs, which are expressed in nanograms per milligram of PM mass. The data show an excellent balance between the total mass and the sum of the measured chemical species, which account for 97% of the total UFP mass. OC is the most predominant species, contributing 41% of the mass. Trace elements and metals, such as Al, Si, Ca, and Fe, are also significant. BGP is the most abundant PAH in the UFP mode.
